# Book Reviews

**Published:** 1902-10

**Authors:** 


### *BOOK REVIEWS.*

---

HARE'S PRACTICAL DIAGNOSIS. Lea Bros. & Co., New York and Philadelphia, 1902.

This is the fifth edition of this well-known work on Physical Diagnosis.

It contains much new material, and the matter in the older editions has been revised and largely rewritten. The book is of inestimable assistance, as the arrangement is such that from prominent symptoms a diagnosis may be constructed by a process of assembling and exclusion. The value of this method may be readily appreciated. The book is profusely illustrated.

---

### LUNG ANTISEPTICS.

The recent announcement of the discovery of an intestinal antiseptic capable of killing all bacterial life in the intestine without injury to the intestine itself is a great step in advance, and if true means the elimination of typhoid fever, dysentery, and other bowel inflammations. If now some scientist would only discover an antiseptic that would exterminate all bacteria in the lungs without injury to the lungs, so that the matter of consumption could be met and the dreadful mortality due to that disease removed, physicians would feel that human agencies were far advanced toward their limits in conquering disease.

Until such a lung antiseptic is discovered physicians must go on, and by purely defensive measures, by nourishing, strengthening, protecting and putting the consumptive's body into the best possible physical condition, try to attain that "power of resistance" which repulses the invasion of disease. Among the potent medicinal agencies for attaining this "power of resistance" cod-liver oil occupies a most prominent position. Scott's Emulsion, the well-known proprietary preparation of cod-liver oil, owes its prominence to the truly remarkable effects of the oil in supplying the consumptive with an easy means of nourishment and a saving of his vitality.

